# Supplementary material for: Desiderata for a biomedical knowledge network: opportunities, challenges and future directions
Source: Bioinform Adv. 2026 Mar 20;6(1):vbag036. doi: 10.1093/bioadv/vbag036 (PMC13004217; doi:10.1093/bioadv/vbag036)
Supplement: vbag036_Supplementary_Data [file vbag036_supplementary_data.zip › Supplemental Figure S1.pdf]

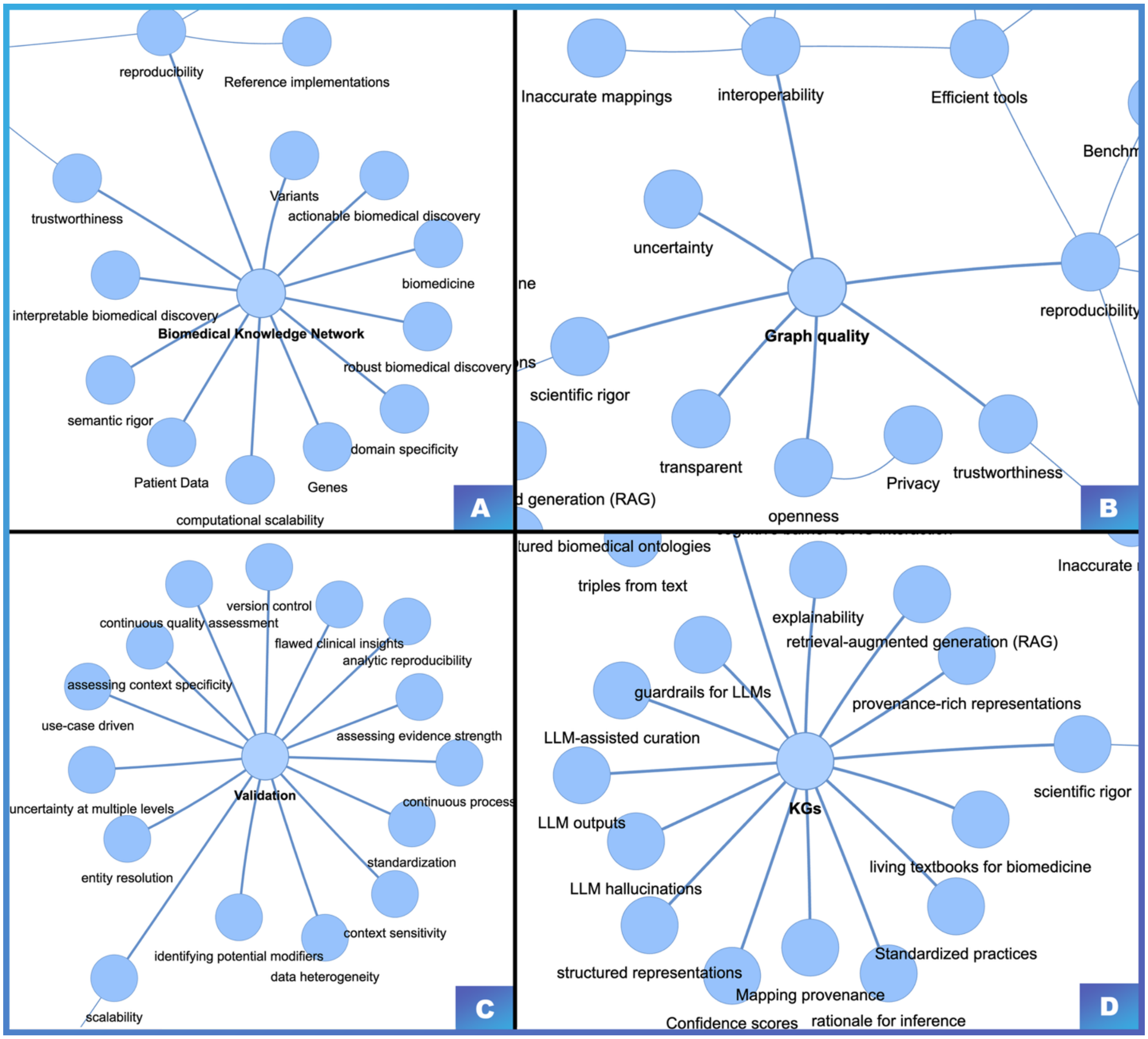

Supplemental Figure S1. Detailed views of key sub-graphs from the Desiderata knowledge graph in Figure 2. Each panel highlights a central hub and its most highly connected concepts, illustrating specific challenges and requirements: **A**) The **Biomedical Knowledge Network** and the complex nature of the domain (e.g., patient data, heterogeneity, actionable discovery,); **B**) **Graph Quality** and critical attributes (e.g., trustworthiness, transparency, interoperability); **C**) **Validation** frameworks (e.g., reproducibility, context specificity, standardization), and **D**) **Knowledge Graphs (KGs)** in the context of emerging methods (e.g., LLM-assisted curation, RAG, hallucinations). The nodes and edges were extracted by processing the Desiderata text using the OpenAI GPT-4 model and rendered as an interactive web page (https://biothings-data.s3.us-west-2.amazonaws.com/knmeeting/graph_desiderata.html) using the vis-network Javascript library (v9.1.2).
