# Supplementary figures and images for: Desiderata for a biomedical knowledge network: opportunities, challenges and future directions
Source: Bioinform Adv. 2026 Mar 20;6(1):vbag036. doi: 10.1093/bioadv/vbag036 (PMC13004217; doi:10.1093/bioadv/vbag036)

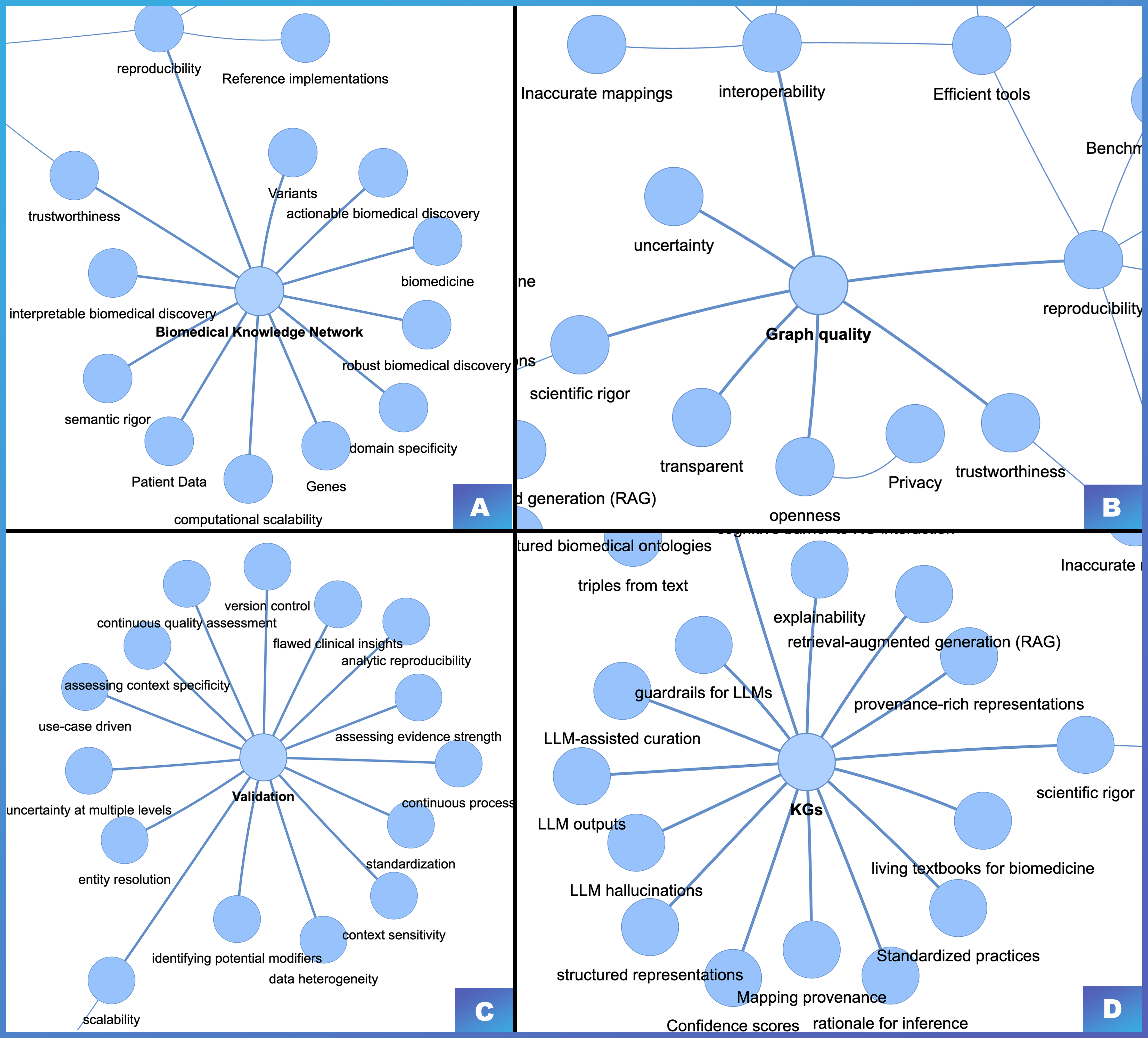

Supplement: vbag036_Supplementary_Data [file vbag036_supplementary_data.zip › Figure_S1.png]
